# The Surrogacy Regulation Act of 2021: A Right Step Towards an Egalitarian and Inclusive Society?

**DOI:** 10.7759/cureus.37864

**Published:** 2023-04-20

**Authors:** Gaurang Narayan, Hara Prasad Mishra, Tarun Kumar Suvvari, Ishika Mahajan, Mrinal Patnaik, Sahil Kumar, Nidhal A Amanullah, Smruti Sikta Mishra

**Affiliations:** 1 Medical Law and Ethics, National Law School of India University, Bengaluru, IND; 2 Obstetrics and Gynecology, Indira Gandhi Government Medical College, Nagpur, IND; 3 Medical Law, National Law School of India University, Bengaluru, IND; 4 Medicine, University College of Medical Sciences, University of Delhi, New Delhi, IND; 5 Computational Medicine, Indraprastha Institute of Information Technology, New Delhi, IND; 6 Pharmacology and Therapeutics, University College of Medical Sciences, University of Delhi, New Delhi, IND; 7 Medicine and Surgery, Rangaraya Medical College, Kakinada, IND; 8 Department of Acute Medicine, Lincoln County Hospital, Lincoln, GBR; 9 Medical Ethics, National Law School of India University, Bengaluru, IND; 10 Forensic Medicine and Toxicology, All India Institute of Medical Sciences Bhopal, Bhopal, IND; 11 Pharmacology and Therapeutics, ESIC Dental College and Hospital, New Delhi, IND; 12 Psychiatry and Behavioral Sciences, Sree Ramakrishna Mission Hospital, Thiruvananthapuram, IND; 13 Occupational Therapy, Pandit Deendayal Upadhyaya National Institute for Persons with Physical Disabilities, New Delhi, IND

**Keywords:** society, ethical dilemmas, human rights and legal issues of health, surrogacy act of 2021, surrogacy

## Abstract

With the advent of major scientific and technological advancements in obstetrics and gynecology, surrogacy is quickly becoming a viable alternative to enable people of all genders to become parents. However, its path toward reality is still fraught with legal and ethical dilemmas. With the Surrogacy Act of 2021 coming into effect earlier this year, the present article aims to dissect the various legal nuances involved while also considering the societal norms governing the actual scenario at ground zero. Our review discusses the aspects of eligibility criteria, the health implications, the rights of the surrogate mother and the child born, the financial burden, and compensation. We aimed to bring attention to this act and its implications on marginalized segments of society, with an attempt to bring beneficial changes for them. In this review, we provide viable alternatives adopted across the globe to solve the identified issues to make the present act non-discriminatory and more rewarding to all involved beneficiaries.

## Introduction and background

Infertility is a growing healthcare concern affecting both men and women. It can result from a variety of factors ranging from age, genetics, lifestyle, and environmental aspects. With the increasing incidence of infertility, there is a need for alternative methods of reproduction to enable people to become parents. Surrogacy has emerged as a viable option due to advancements in artificial reproductive procedures [1]. In addition to infertility, changes in societal norms have also played a role in the acceptance of surrogacy. In today's progressive society, both men and women recognize infertility as a healthcare problem, and parenthood is no longer restricted to the heterosexual community. Advancements in artificial reproductive procedures have enabled people of all genders to become parents, with surrogacy emerging as a viable alternative [1,2].

The term "surrogacy" refers to the practice of using a woman's womb to carry a fetus until birth to be raised by another [2]. It is derived from the Latin word "subrogate", meaning "accepted to act in the place of" or "a substitute". As per American Law Reports, surrogacy is typically defined as “...a contractual undertaking whereby the natural or surrogate mother, for a fee, agrees to conceive a child through artificial insemination with the sperm of the natural father, to bear and deliver the child to the natural father, and to terminate all of her parental rights after the child's birth” [3]. 

Surrogacy can be classified into altruistic and commercial. True to the meaning of the word, altruistic surrogacy entails no financial compensation for the surrogate. In contrast, commercial surrogacy involves paying the surrogate for bearing the child, implying a profit, while compensated surrogacy simply involves covering the incurred expenses and loss of wages [4].

Commercial surrogacy was legal in India between 2002 and 2015. During this period of legalization, the "businesses of commercial surrogacy flourished in lieu of the vast number of underprivileged women eager to make a fair living by renting their wombs" [5]. Unfortunately, this need was capitalized on by middlemen, who created a nexus between the healthcare system and women, resulting in the exploitation of the latter. In 2012, the annual turnover of this surrogacy market was estimated to have been worth as much as 2.5 billion USD [6]. There are no clear data on the number of verified couples who sought out surrogate mothers in India during this legalization era. Estimates state that of the approximately 25,000 surrogate children born in India every year, at least 50% were for couples from the Western world [7]. A surge in the number of cases of procreative medical tourism has been noted in recent years. Due to the wide accessibility of affordable state-of-the-art therapies for assisted reproductive technologies, India is the go-to destination for surrogacy [8]. 

As surrogacy gained popularity across the globe, distinct legal regulations began to be crafted in different countries. Complex legal difficulties emerged as the framework in each country aimed at different outcomes. The regulations were so vast and varied, irrespective of their intent, whether to promote, regulate, or ban surrogacy. While countries such as the USA, Georgia, Ukraine, and Colombia have surrogacy-friendly legislations, restrictive regimes have been imposed in Iceland, Germany, Sweden, Austria, and others. Consequently, nationals from countries that have banned surrogacy turned to commission it overseas, resulting in statutory difficulties when the laws clashed across international borders [9]. Additionally, this legal clash also raised concerns about evasive travel [10]. From the perspective of increased demand, this influx of cases into India, in the background of a lack of regulation and international coordination, paved the way for unethical practices and exploitation.

Due to the wide financial gap and the disparity between individuals engaged in the practice of commercial surrogacy, both the surrogate and the child were vulnerable to exploitation [11]. Numerous incidents of harassment of surrogate mothers were reported to the police in 2018-2019. Human rights exploitation rackets in the guise of surrogacy were exposed and arrests were made in 2019 [12]. This revealed an urgent need for an expediting of regulations to be placed on surrogacy. The increasing demand and the unscrupulous activities resulting in the ill-treatment of vulnerable groups forced the Indian government to take action and propose the Surrogacy Regulation Bill of 2015. A need for guidelines required to protect the commissioning parents' rights was also raised at the same time.

The issue was raised in the Lok Sabha of the Indian Parliament when the government took a stand to disallow commercial surrogacy in its response to question 100 on 4th December 2015. This led to the creation of the Surrogacy (Regulation) Bill in 2016, which, following multiple amendments, was passed in 2018 by the Lok Sabha. The Rajya Sabha created a committee for discussion of the Surrogacy (Regulation) Bill 2019 with various stakeholders, the conclusion of which led to some more amendments, culminating in its passage into law on December 25, 2021. It was released along with the Assisted Reproductive Technology (Regulation) Act, 2021, just a week prior. On January 25th, 2022, the new Surrogacy (Regulation) Act, 2021, went into force. The amended act exclusively permits charitable surrogacy, preventing those with financial means from abusing and taking advantage of the surrogacy option. It prohibits commercial surrogacy, as well as the trade of human gametes and embryos [4,12].

## Review

The Surrogacy Regulation Act, 2021: highlights

Intending Couple, Intending Woman, and Surrogate Mother: Definitions and Eligibility

Chapter 1 of the 2021 Act identifies the most significant parties involved in gestational altruistic surrogacy. Chapter 3 establishes the requirements for them to be eligible for altruistic surrogacy in the Indian subcontinent.

An "intending couple" is an Indian infertile married couple, per the Act (the age of the woman being 23 to 50 years and the age of the man being 26 to 55 years). The couple must not have any living children in order to receive a certificate of eligibility for surrogacy (biological, adopted, or surrogate). The only circumstances in which this clause would not apply is if their surviving child has a disability, either mental or physical, or if the child has a condition that poses a serious risk of death [4,12,13]. The Act also permits Indian widows, divorcees, and married couples of Indian origin living abroad to become parents through altruistic surrogacy. An Indian widow or divorcee between the ages of 35 and 45 who plans to use surrogacy is referred to as an "intending woman" [4,12,13].

In the new law, the definition and requirements for becoming a "surrogate mother" have been updated as follows:

a) Any willing, ever-married woman between the ages of 25 and 35 who has her own child may become a surrogate (does not address her eligibility should this child be borne of surrogacy itself). b) May only sign up for surrogacy once in her lifetime, but up to three attempts may be undertaken if embryo transfer does not take place. c) Must be physically and mentally fit, as attested by a medical practitioner through certification. d) Prohibited from providing her own gametes for surrogacy by the Act. e) Not receive any compensation for carrying the child in her womb other than the necessary insurance and medical costs. f) For a period of 36 months, insurance must cover any difficulties arising from the delivery of the baby, including postpartum complications and even death [4].

In addition, the surrogate mother has the choice to revoke her participation even right up until the embryo is placed in her womb and should a need arise, even to terminate the pregnancy, as per the Medical Termination of Pregnancy Act, 2019 [4,12,13].

Prerequisites for surrogacy as per the Act

As per the Act, only those cases fulfilling the following situations would qualify for the use of surrogacy procedures when there is a medical indication. The District Medical Board must issue this indication certificate in favor of the commissioning party when [4]:

a) The intended parents are of Indian origin. b) The intended mother is a divorcee or widow. c) The surrogacy is for charitable purposes. d) It is not being done for financial gain

The Surrogacy Regulation Act, 2021 - threats to the ethical, social, and legal constructs - critical reflections: the ethos of Indian society: implications of coercion and views on death

Depriving Plebeians of Their Reproductive Autonomy

To avoid the exploitation of women, the current Act maintains strong checks and balances. It makes an effort to cut out the predatory middleman. It safeguards and upholds the value of motherhood as well as any prospective parental rights over the child. However, the outlawing of commercial surrogacy shifts the focus from a right-based approach to a need-based one. The decision pertaining to whether or not one can have children, and the number of children they want should rest with the individuals themselves and not the government. It is offensive and only reflects conservative ideas to base this Act's principles on compensation.

The recent Act also prohibits the following groups from utilizing surrogacy services:

Couples with one child, foreign nationals, in-residence partners or people in "live-in relationships", single men and women, gay and lesbian couples, and widowers

While the Act does not explicitly introduce any gender bias when referring to the child so born, it does not address the finer intricacies of an already complex parentage. By including this language, the regulating Act criminalizes surrogacy in socially oppressed communities and creates the foundation for a patriarchal and heteronormative society.

Glass ceiling "women’s rights"

Unmarried pregnant women are legally permitted to undergo abortions, if they wish to do so, under the 2021 amendment of the Medical Termination of Pregnancy Act, 1971 [14]. While this Act recognizes a woman’s rights to her own body, the surrogacy Act prevents unmarried women from availing of the services of surrogacy. Although there have been some instances of human rights abuse of women in the setting of commercial surrogacy, there have also been examples where it provided them with a dignified life, financial independence, and even the opportunity to educate their children in an attempt to secure their future [15]. A woman’s reproductive choice is a fundamental right and is an indissoluble segment of her freedom and liberty as enshrined under Article 21 of the Indian Constitution [16]. Using the social construct of marriage to determine eligibility for surrogacy and become a surrogate hinders reproductive autonomy and confines the beneficial provisions to a section of society [17]. Furthermore, regarding the modification to the definition of reproductive rights, setting an age limit for women to become either surrogate mothers or intending mothers denies them their basic reproductive rights.

Surrogacy and the LGBTQIA+ community

The 2021 Act bars homosexual couples from using altruistic surrogacy. It plays into a stereotyped view of a family, not only in a heteronormative household dynamic but also presuming a lack of autonomy on the part of women. Indian law, societal standards, and religious doctrine declare that both parents must be from two different sexes for the holistic development of a child. In contrast to this mindset, the hypocrisy in this idea is founded on the fact that it permits the utilization of ART services by widowed and divorced women. This is discriminatory and excludes the LGBTQIA+ community from the purview of surrogacy [12].

Interestingly, one can still claim that the Act adheres to the Universal Declaration of Human Rights, adopted in 1948 and ratified by India. Article 16.1 of the Declaration states that "men and women of full age without any restriction due to race, ethnicity or religion have the right to marry and have a family" [18]. The Indian judiciary, conscious of this, regards the right to procreate as a fundamental one. For instance, the Andhra Pradesh High Court recognized the civil rights to rightly include the freedom to reproduce and affirmed that "the right to reproductive autonomy" is inclusive under the "right to privacy" in B. K. Parthasarthi v. Government of Andhra Pradesh [19].

In the Navtej Singh Johar v. The Union of India case, the Supreme Court of India unanimously declared that Section 377 was unconstitutional [20]. The learned judges presiding on this case proclaimed that to "attack" the LGBTQIA+ community on account of their sexual orientation is against the fundamental rights to equality, freedom of speech, right to choose, and the right to dignity. The LGBTQIA+ community was also assured to be entitled to equal legal rights and to be treated equally in society without experiencing any stigma [20].

Through surrogate arrangements in India, many gay couples from Spain and Israel were successful in starting families [21,22]. This provides evidence that parenting a same-sex child is the same as raising a child of a heterosexual marriage. Due to the effective surrogacy ban in the region, there has been a marked surge of single gay men or gay couples as well as single lesbian females or lesbian couples choosing to become parents in Mexico or the US [23,24]. Although decriminalizing homosexuality was a welcome step, revolutionary constitutionalism and its incorporation into public policy still have a long way to go. Despite the repeal of Article 377, the LGBTQIA+ population continues to face societal stigma and denial of fundamental civil rights [12,13]. In the landmark decision of the National Legal Services Authority v. Union of India, the Supreme Court recognized transgender people as a third gender [21]. The 2021 Act makes no mention of granting the people belonging to the third gender equal rights.

It is therefore safe to infer from the aforementioned legal precedents that the courts have upheld physical and sexual autonomy by decriminalizing Section 377 under Articles 14, 19, and 21. It is regrettable that the same policy has not been implemented to permit members of the LGBTQIA+ community to become parents through surrogacy.

Excluding live-in relationships from the scope

Live-in partners are not covered by the Act's regulatory scope. Contrary to popular belief, live-in relationships between consenting adults are not against the law in India. In the 2006 case of Lata Singh v. State of UP, the same was upheld [25]. In S. Khushboo v. Kanniammal, the Supreme Court ruled that a live-in relationship is covered and protected by Article 21 (right to life) [26]. In Badri Prasad v. Director of Consolidation, the Supreme Court upheld the legality of a 50-year live-in partnership [27]. The Allahabad High Court stated in Payal Sharma v. Superintendent. Nari Niketan, that "a man and a woman, even without getting married, can live together if they choose". The courts have thus made it clear that not all socially unacceptable behavior must be deemed unlawful [28]. However, why the right to choose the path of parenting is not extended to these couples is unclear and remains to be addressed.

The phrase ‘husband" has been replaced with "partner" in the most recent Medical Termination of Pregnancy Amendment Act of 2021, which also grants any woman with an unintended pregnancy the right to use abortion services [29], regardless of her marital status. Therefore, it is tantamount to hypocrisy when Indian courts approve of live-in relationships, but instead of considering the merits of a legal question on such an issue, they would rather ponder if it is likely to "promote" the concept of "promiscuity", and insulting too when such promiscuity is de facto associated with unmarried women. The Act's use of the same theory to limit who may serve as a surrogate is also unfair.

Altruistic model promoting "forced labor"

The pressing concern in the House is that the prevalence of illicit or covert surrogacy will rise as commercial surrogacy is outlawed. Through the use of unethical and corrupt techniques, the infertility healthcare industry’s common desire to maximize profits will further pave the way for exploitation and corruption [4,9,10,12]. A recent incident of a minor girl allegedly being forced to sell her eggs for donation is a classic example of the nefarious dealings that can occur [30].

Article 19(1) is violated if commercial surrogacy is entirely prohibited. The poverty that already exists and other pressing needs encourage forced labor. Putting restrictions on the bodily autonomy of consenting adults' right to earn a livelihood should not come at the cost of human dignity, which a commercial racket seemingly exploits. Therefore, it is essential to reach a compromise that supports commercial surrogacy under an umbrella of legislation to protect human rights. The idea of "compensatory surrogacy" can be explored, where the costs could be established by qualified authorities [31,32].

Long-term health implications on the surrogate mother and the fetus: an unsettled debate

Teratogen and maternal drug abuse have always been a matter of concern for the health of the fetus. If in any case, the fetus is affected due to an accidental or intentional overuse of drugs by the surrogate, the rules and regulations say nothing about the consequences for her [12,33]. The current Act also fails to shed light on the possible risks of venereal diseases the mother might contract and the risk of these infections affecting the fetus in utero. Extensive screening of intending parents is a seemingly possible solution to prevent such mishaps from occurring [34,35].

Several studies also report a significantly increased incidence of postpartum consequences in surrogate pregnancies. Commonly encountered maternal complications include gestational diabetes mellitus, hypertension complicating pregnancy, and the risk of postpartum hemorrhage. Considering that surrogacy is associated with the technique of IVF where generally more than one embryo is implanted, there is a risk for the fetus related to multiple gestations, low birth weight, and preterm delivery [36]. Providing mere insurance coverage for a period of 36 months may not help the surrogate mother [12,37]. The current Act fails to anticipate health problems concerning the surrogate mother and the child.

Unseen mental health implications

While the current Act is comprehensive and very stringent in terms of determining the psychological eligibility of a surrogate, it fails to shed light on the possibility of new-onset psychiatric disorders post-implantation. Furthermore, unaddressed mental health issues in the surrogate mother might pose dangers to the fetus in utero [38,39]. The current Act only speaks of ensuring psychological fitness while screening and approving surrogate mothers. Although such a provision exists, the Act does not specify a formal psychiatric assessment by a mental health practitioner for the purposes of a psychological fitness certificate. This reflects the ignorance of legal bodies towards addressing possible mental health issues for holistic healthcare delivery [40,41].

Furthermore, it cannot be ignored that having a surrogate baby is associated with various short-term and long-term mental issues. Several studies highlight the possibility of the surrogate mother developing postpartum depression, postpartum blues, or postpartum psychosis. Catering to the emotional and mental dimensions of health becomes extremely important for the well-being of the child. These lacunae need to be effectively addressed [34,35,42].

Discrimination on the basis of "ableism"

As per the new Act, a couple with a child diagnosed with an incurable physical/mental illness (as duly approved by the District Medical Board) can avail of the services of surrogacy. There is, however, an unclear and ineffective guideline to define the limits of such disability and the various conditions it encompasses. This creates a lacuna in understanding the extent of disability and determining the degree of dependence. Hence, the current algorithm is inadequate, as it does not specify and elucidate the physical/mental disability, thereby discriminating against the common man on the basis of ableism [43].

On the other hand, on the arrival of a new healthy child, the disabled child may face the risk of being emotionally and financially neglected. While welcoming a second child can promote interaction and emotional bonding with the first child, forming an effective support system can be tricky, especially if the second child feels it to be forced [44, 45].

The downfall of procreative medical tourism

When surrogacy was fully legal in India, advancements in surrogacy led to the rise of "procreative medical tourism". The low treatment cost and availability of modern reproductive techniques make India the best destination for infertility treatments (6,7,10). After the landmark cases Baby Manji Yamada vs. Union of India and Jan Balaz vs. Anand Municipality, the provisions for surrogacy concerning the contracts of custodianship and citizenship of the children born to intending foreign nationals were scrutinized [46]. Keeping in mind the agenda of protecting the child’s rights, the current Act was designed to ban foreigners from availing of the services of surrogacy in India. This in turn led to a glut in the massive flock of "fertility tourists" to India [41,44].

Although the current Act is a step toward protecting the child’s rights, it completely neglects the economic aspects and financial compensation for the surrogate mother. India suffers from poverty, and with the advent of surrogacy in the country, it was seen as a possible solution to help women from poor backgrounds find a job. In this altruistic model, there is no economic advantage for women from poor backgrounds, and with the above-mentioned examples of forced labor and covert surrogacy, the surrogate women fail to reap economic gains. Thus, rather than completely banning commercial surrogacy, finding alternatives that strike a balance would help provide an effective solution to tackle the risk of exploitation of poor surrogate mothers [45,47].

Short-term disability insurance coverage: a seemingly possible solution for the compensation of lost wages

Short-term disability (STD) insurance coverage is a program that provides early financial assistance to people with disabilities to help them continue working. It avoids the provision of long-term benefits with the retention of a modest compensation [48]. This particular provision was recently extended to surrogates to make up for some of their lost wages. Although this insurance does not typically compensate for all the lost income of the surrogate, it will still be of some help to them. It is not clear whether they would be eligible to avail themselves of the maternity benefits that allow for 26 weeks of paid maternity leave. In such situations, this provision of short-term disability insurance coverage as available in parts of the US may be a viable solution [49,50].

In addition to paying for the health coverage of the surrogate, to prevent ethical problems unrelated to payments, additional financial funding may be needed. Some global models suggest provisions for independent legal representation of the surrogate to ensure adequate monetary compensation for the financial losses sustained and the possible mental and physical health issues that may arise in the future [51,52].

## Conclusions

While the Surrogacy (Regulation) Act of 2021 attempts to regulate surrogacy in India, it falls short of meeting the Golden Triangle Test of protecting fundamental rights guaranteed by the Indian Constitution. The Act effectively excludes certain sections of society, such as LGBTQIA+ individuals, from opting for surrogacy as a last resort for biological parenthood. Additionally, the Act places numerous hurdles in the name of protecting women from the pitfalls of commercial surrogacy and the artificial reproduction industry, which could have unintended consequences.

Therefore, there is a need to amend the Act to make it a more comprehensive piece of legislation that supports an inclusive and egalitarian society. This can be achieved by acknowledging the shortcomings, addressing the problems, and ultimately arbitrating the law to strike a balance between the interests of all stakeholders. The new laws and regulations must also take into account changing societal patterns to ensure that they remain relevant and effective in addressing the concerns surrounding surrogacy while protecting the fundamental rights of the individuals.
